# Beyond liver cancer, more application scenarios for alpha-fetoprotein in clinical practice

**DOI:** 10.3389/fonc.2023.1231420

**Published:** 2023-09-15

**Authors:** Chenyu Ma, Yuexinzi Jin, Yuhan Wang, Huaguo Xu, Jiexin Zhang

**Affiliations:** ^1^ Department of Laboratory Medicine, The First Affiliated Hospital with Nanjing Medical University, Nanjing, China; ^2^ Center of Smart Laboratory and Molecular Medicine, School of Medicine, Chongqing University, Chongqing, China

**Keywords:** alpha-fetoprotein, clinical application, tumor, pregnancy, fetal diseases

## Abstract

Alpha-fetoprotein (AFP) is a commonly used clinical biomarker. Before 1970, the two-way agar diffusion method was mainly used, and the specificity of AFP in the diagnosis of primary liver cancer was satisfactory. However, its positivity rate was not very high. The diagnostic value of AFP is changing with the evolution of detection methods. Here, we performed a literature search to identify English-language publications. The search was performed from January 2015 to April 2023 using the PubMed database and the following terms in [Titles/Abstracts]: alpha-fetoprotein, clinical practice, detection, etc. The references of retrieved articles were also screened to broaden the search. Studies referring to liver cancer and AFP detection methods were excluded. In this review, several clinical application scenarios for AFP were systematically reviewed, and its potential detection value in the future was discussed.

## Introduction

Alpha-fetoprotein (AFP) is a glycoprotein and has a total of 591 amino acids (molecular weight: approximately 70 kDa). It belongs to the albumin family and was originally found in human fetuses, where it is produced by embryonic hepatocytes, yolk sac cells, and fetal gastrointestinal cells (1). Serum AFP levels rapidly decrease after birth, and its synthesis is virtually absent under normal physiological conditions. In clinical practice, serum AFP is widely acknowledged as a surveillance and diagnostic biomarker for hepatocellular carcinoma (HCC) (2).

Nevertheless, a portion of HCC patients (approximately 30%-40%) are diagnosed with AFP-negative HCC (AFPN-HCC) (3). Serum AFP levels are not always related to tumor burden which is characterized by an atypical fluctuation within its reference range in advanced HCC before proper clinical treatments (4). Liu et al. enrolled 597 patients with viral hepatitis B-associated liver disease as well as 201 healthy controls and established a nomogram model with outstanding significance consisting of multiple serum indexes and clinical parameters for the diagnosis of AFPN-HCC (5). It helps to release from the dilemma of relying solely on imaging examination in AFPN-HCC patients. It also raises the possibility that other candidates are expected to replace AFP as ideal surrogates for HCC (6).

The application of AFP is further limited by its inadequate specificity due to serum elevation in ALT flare in CHB and liver cirrhosis (7). Yu et al. analyzed serum markers of hepatocyte destruction and regeneration in 103 patients who diagnosed with chronic hepatitis type B (CHB) and described a model-based quantitative relationship between serum AST and AFP in distinguishing CHB patients with remission from those with progression (8). The so-called “host-dominating flare” characterized by substantial fluctuation of AFP accelerates hepatitis B virus DNA elimination, surface antigen decline, and favorable treatment response. Moreover, there are still some controversies, and several cases have been reported to describe the involvement of AFP in the occurrence or development of other diseases. In this review, we will summarize more application attempts of AFP in non-HCC diseases and discuss its clinical value from aspects of sensitivity, specificity, and clinical relevance.

## Tumors

### Gastric cancer (GC)

GC is the fifth most common malignancy worldwide and the third leading cause of cancer-related deaths (9). The unsatisfactory prognosis is mainly due to the advanced stage at diagnosis (10). AFP-positive gastric cancer (AFPP-GC) is a rare entity, the incidence of which is reported to be 1-15% among GC patients (11). AFPP-GC presents an aggressive behavior, leading to high recurrence rates and short postoperative survival (12). Reim et al. conducted a study that included 3,034 GC patients who underwent curative surgery without any residual disease between 2002 and 2007 (11). A total of 97 patients were AFP-positive [a preoperative serum AFP level >10 μg/L or positive immunohistochemical (IHC) staining]. Propensity score matching (PSM) analysis and multivariate models were used to reduce bias, and 87 AFPP-GC patients were matched to the same number of AFP-negative GC (AFPN-GC) patients. They found that only serum AFP elevation, age, and pathological tumor stage were predictive for overall survival (OS) (p < 0.0001). Recurrence-free survival (RFS) was worse in AFPP-GC patients (p = 0.002). Compared to that in AFPN-GC patients, the recurrence rate was higher (34.5% vs. 16.9%, p = 0.003), the five-year survival rate was remarkably lower (57.9% vs. 76.1%, p = 0.014), and the five-year RFS rate was significantly lower (53% vs. 76%, p = 0.0029) in AFPP-GC patients. The author proposed that AFPP-GC should be considered a distinct clinical entity. Preoperative serum AFP elevation (>10 μg/L) is a poor independent prognostic predictor for GC. Frequent follow-up and active adjuvant therapy help to reduce the possibility of recurrence after radical gastrectomy.

### Colorectal adenocarcinoma with enteroblastic differentiation (CAED)

CAED is a rare subtype of colonic adenocarcinoma, and it often appears in the sigmoid colon or rectum with metastasis in lymph nodes and distant organs (13). Murakami and colleagues identified five CAED patients (0.3%) from 1,666 patients with colorectal carcinomas whose clinical pathological features were analyzed followed by IHC staining with enteroblastic lineage markers: glypican-3 (GPC-3), spalt-like transcription factor 4 (SALL4), and AFP (14). Four patients were positive for SALL4 and GPC-3, and one patient had an AFP-positive hepatoid component, indicating that AFP, GPC-3 and SALL4 are characteristic of CAED. Abada et al. further confirmed the coexpression pattern of AFP, GPC-3, and SALL4 in liver metastatic lesions from a 26-year-old female diagnosed with CAED (15). However, the pathological value of intercellular AFP is still unclear because of the limited number of CAED patients.

### Xp11 translocation renal cell carcinoma (RCC)

RCC is a mesoderm-originating malignant tumor, and AFP-producing RCC has been occasionally reported since 1988 (16). Recently, Zhang et al. reported a male patient with a renal mass in the lower pole of the left kidney and normal serum levels of carbohydrate antigen 199 and carcinoembryonic antigen (17). His serum AFP level was higher than 3,000 ng/mL and gradually decreased to 13.54 ng/mL two months after radical nephrectomy. Postsurgery fluorescence *in situ* hybridization (FISH) showed rearrangement of the *transcription factor binding to the IGHM enhancer 3 (TFE3)* gene, which is the specific marker of Xp11 translocation RCC. IHC examination of the resected lesion showed diffuse positivity for AFP, GPC-3, and TFE3. Another case of AFP-producing Xp11 translocation RCC was reported by Shim et al. in a 51-year-old woman (18). She had a multiseptated cystic mass in the right kidney, and her serum AFP level was 313.3 ng/mL. After right partial nephrectomy, the serum AFP level dropped to 13.5 ng/mL. The tumor cells were diffusely and strongly positive for AFP, and they exhibited hepatoid features with alveolar, acinar, or microcystic patterns. The two reports raise attention to the universality of AFP, which reflects progressed dedifferentiation or retrodifferentiation of RCC cells as a result of the complexity of genomic instability and tumor heterogeneity (19).

### Urothelial carcinoma (UC)

UC is the most common type of bladder cancer with a significant risk of mortality and recurrence (20). The estimated lifetime morbidity of UC is reported as 1 of 25 in men and 1 of 80 in women in the United States (21). The five-year survival rate for patients with metastatic UC is less than 10% (22). UC can be treated effectively with immunotherapy because of its high rate of somatic mutations and high antigenicity (23). Programmed death-1 (PD-1) and programmed death ligand 1 (PD-L1) checkpoint inhibitors have emerged as new treatment options for metastatic UC. Pembrolizumab and nivolumab are PD-1 inhibitors. Atezolizumab, durvalumab, and avelumab are PD-L1 inhibitors that have been approved by the FDA for refractory metastatic UC (23). Melms et al. demonstrated a case of chemoresistant metastatic UC and chronic hepatitis B in a 62-year-old patient (24). The serum AFP level of the patient was 934.7 ng/mL, and it sharply declined after radical cystoprostatectomy and pelvic lymphadenectomy. IHC examination of the primary lesion showed strong staining for AFP and PD-L1. The patient experienced significant recurrence and progression with re-elevation of the serum AFP level to 3,800 ng/mL after three months. Considering the history of long-term chronic hepatitis, the patient was treated with pembrolizumab (an anti-PD-1 checkpoint inhibitor). The serum AFP level decreased to 42 ng/mL within six weeks, which coincided with the resolution of all palpable metastatic lesions. After 16 cycles of pembrolizumab, the patient showed complete clinical remission, and the serum AFP level was less than 5 ng/mL. The author highlighted the application of AFP as a marker for anti-PD-1 therapy response and proposed its value in UC progression surveillance.

### Recurrent testicular germ cell tumor in adult

According to data from the UK and US Office for National Statistics on Cancer registries, approximately 60% of testicular tumors are seminoma germ cell tumors (SGCTs), and the rest are mainly nonseminoma germ cell tumors (NSGCTs) (25). Testicular cancer is curable, and the long-term survival rates are high for most patients (5-year survival rate > 95% in the US, 10-year survival rate > 98% in the UK) (25). The AFP decline rate is an important prognostic factor for NSGCT in adults (26). As European guidelines recommend, three biomarkers, β-human chorionic gonadotropin (β-hCG), lactate dehydrogenase (LDH), and AFP, should be measured on schedule according to treatment protocols and tumor stage (27).

Nicholson et al. systematically reviewed the data for the diagnostic accuracy of serum β-hCG, LDH, and AFP in monitoring testicular cancer recurrence in adults (25). The specificity of AFP alone is generally high (~100%) in the recurrence diagnosis of SGCTs and NSGCTs. However, the sensitivities of AFP were 36%, 100%, and 86% in two NSGCT studies and one study of both SGCTs and NSGCTs, respectively. When a combination of AFP and β-hCG was used, the estimated specificities were relatively stable (>95%), and the estimated sensitivities ranged from 36% to 100% according to different research teams’ reports. Nicholson considered that the recommendation of β-hCG, LDH, and AFP for monitoring testicular cancer recurrence during follow-up in international guidelines is lacking in supporting evidence. The involvement of LDH in recurrence is not yet clear. Upcoming cohort-based studies should explain the appropriateness of biomarkers, biomarker cut-points, and testing intervals to establish a follow-up strategy.

### Childhood NSGCTs

Fresneau et al. studied the prognostic value of serum AFP levels for childhood NSGCTs in a French TGM95 study (a prospective nonrandomized study conducted for patients aged ≤ 18 years with extracranial NSGCTs between January 1995 and December 2005) (28). A total of 239 patients were included [114 patients with high risk (serum AFP levels ≥ 15,000 ng/ml and/or metastatic tumors), 65 patients with medium risk (localized incompletely resected tumors, serum AFP levels < 15,000 ng/ml), and 60 patients with low risk (localized and completely resected tumors, serum AFP levels < 15,000 ng/ml)]. The main lesions were in the ovary (n = 77), testis (n = 66), and sacrococcygeal region (n = 57). The five-year OS and progression-free survival (PFS) rates were 93% [95% confidence interval (CI): 89-95%] and 85% (95% CI: 80-89%), respectively. The analysis of AFP decline indicated that AFP change (p = 0.10) and predicted time to normalization (p = 0.61) were not prognostic, in contrast to those in adult NSGCTs. The effect of differential observed and expected AFP decline area under the curves (O-E AUCs) was significant (HR = 2.1, 95% CI: 1.0-4.2, p = 0.05). A higher risk of progression/recurrence was observed in patients who showed slower serum AFP decline. PFS was significantly poorer when the difference between O-E AUCs increased.

### Neonatal sacrococcygeal teratoma (SCT)

SCT is the most common congenital tumor, occurring in approximately 1 in 27,000 live births, and the proportion of female patients is higher than that of male patients (29, 30). SCT can be treated by complete resection and removal of the tailbone. Benign SCT can transform to malignant SCT with advanced age or surgical resection (31). Ultrasounds, MRI imaging studies and serum tumor markers are of value during postoperative follow-up (32). AFP is an important marker for SCT (33). A prolonged half-life (HL) and elevated levels of serum AFP are among the predictors of recurrence (34).

Nam et al. studied 55 neonates with nonrecurrent SCT and 10 neonates with recurrent SCT who underwent surgical treatment between 1997 and 2016 (35). Serum AFP levels were detected more than three times in each patient. In nonrecurrent SCT patients, serum AFP HL and age were positively correlated using the formula T_HL_= 0.0597 × days + 6.1643 (p < 0.001). This correlation significantly differed (p < 0.05) from that in recurrent SCT patients (T_HL_= 0.1196 × days −0.0633, p < 0.001). Of 55 neonates with SCT, 41 tumors were mature, and 14 tumors were immature. Serum AFP HLs were differently calculated in immature SCT (T_HL_= 0.0433 × days +8.9339, p = 0.003) and mature SCT (T_HL_= 0.0671 × days + 4.3912, p < 0.001). However, this study had some limitations. For instance, the comparison of AFP HLs between neonates with and without SCT was not included, and the measurement intervals of serum AFP were not controlled.

### Nuclear protein in testis (NUT) midline carcinoma

NUT midline carcinoma is a rare type of squamous carcinoma and is one of the most aggressive solid tumors in humans (36, 37). NUT rearrangement is thought to initiate tumorigenesis (38). NUT fusion proteins are important in NUT midline carcinoma, as they cause growth arrest and irreversible squamous differentiation (39). Nevertheless, the detailed molecular mechanisms of NUT midline carcinoma remain elusive, and surveillance markers are being explored (40). D’Ambrosio et al. reported a case of a 22-year-old man who had a large mediastinal mass with lymph node and skeletal metastases (41). He had life-threatening superior vena cava syndrome, and his serum AFP level was 765 ng/mL. After chemotherapy with cisplatin and etoposide for two cycles, the serum AFP level dropped to 505 ng/mL, which was consistent with the attenuation of clinical symptoms. The serum AFP level rebounded to ~700 ng/mL after 1.5 months when the disease progressed. The author proposed the correlation of serum AFP with disease course.

## Gynecological and obstetric diseases

### Teratomas

Teratomas belongs to NSGCTs and can be classified into malignant immature solid lesions and benign mature cystic lesions (42). AFP is an important marker for the clinical differentiation between benign teratomas (within the normal range) and malignant teratomas (higher than the normal range) (43). Nevertheless, Caposole et al. described a case of metachronous bilateral recurrent ovarian and mediastinal teratomas with slightly increased serum AFP levels (12.3 μg/L) (44). After a multidisciplinary consultation, the patient underwent a right video-assisted thoracic resection, and the lesion was identified as a benign cystic teratomas. After follow-up for six months, the patient showed good recovery. However, her serum AFP level was still high (12.9 μg/L).

### Pathological placentation

Pathological placentation is defined as an abnormal location of the placenta, abnormal invasion of the placenta into the uterine wall, or both (45). The incidence of placenta accreta has been increasing from 1 in 30,000 pregnancies in the 1960s to 1 in 300 pregnancies in more recent reports (46, 47). The incidence of placenta previa was estimated to be 1 in 200 pregnancies worldwide (48). In a retrospective case-control study at Helen Schneider Hospital for Women and Lis Maternity and Women’s Hospital conducted from 2007 to 2014, a total of 307 deliveries were divided into four groups: placenta accreta (64 patients), placenta previa (66 patients), both placenta accreta and placenta previa (17 patients), and normal placentation (153 patients) (49). Berezowsky et al. measured and compared serum biochemical markers between 16 + ^0^ and 19 + ^6^ weeks of gestation in all groups. The group with placenta accreta or placenta previa had a higher median AFP and β-hCG multiples of median (MoM) than the normal placentation group. The highest values of β-hCG (1.59 MoM) and AFP (1.19 MoM) were observed in the placenta accreta group, whereas the lowest values were observed in the control group. The results further showed that the AUC of AFP was 0.573 (95% CI: 0.515-0.630, p < 0.0274), and a cutoff value above 0.99 MoM for the prediction of pathological placentation demonstrated a specificity and sensitivity of 46% and 71%, respectively. The AUC of β-hCG was 0.662 (95% CI: 0.605-0.715, p < 0.0001), and a cutoff value of 1.25 MoM indicated a specificity and sensitivity of 68% and 53%, respectively. The AUC increased to 0.668 (95% CI: 0.611-0.721, p < 0.0001) with 64% specificity and 63% sensitivity when both markers were included. A high-risk group with a 2.27 odds ratio (OR) (95% CI: 1.42-3.63) for pathological placentation and a low-risk group with a 0.38 OR (95% CI: 0.24-0.60) for pathological placentation could be distinguished by the 75^th^ percentile and 25^th^ percentile MoM cutoff of AFP or β-hCG or both. The author suggested that serum AFP and β-hCG can be used as second trimester biomarkers to determine the risk of pathological placentation.

Yu et al. reported a young patient who had central placenta previa accompanied by intermittent bleeding (50, 51). A gradually enlarging hypoechoic area between the placenta and the uterine wall was confirmed by gynecological sonography. She presented with an extremely high AFP level (1,032 ng/ml) in her late pregnancy. Prenatal peripheral blood tests also showed high levels of serum C-reactive protein and neutrophils. During cesarean section, placental necrosis was confirmed by the naked eye. The serum AFP level returned to normal before the patient was discharged. Placental separation from the uterus led to barrier leakage, which increased the amount of AFP in the maternal serum. However, the relationship between the degree of placental damage and AFP has not yet been studied. The author suggested that the combination of AFP and ultrasound imaging may be a valuable tool to assess the status of the maternal-fetal interface.

### Early pregnancy loss

AFP restrains maternal immune responses against the fetus to avoid pregnancy loss (52). It is well acknowledged as an important reference index for the early detection of chromosome malformations and monitoring fetal development (53, 54). Mor et al. investigated AFP as a candidate biomarker for intrauterine pregnancy (IUP) failure (55). There were 78 women in four groups: 1) missed/incomplete miscarriage; 2) threatened miscarriage; 3) cerclage; and 4) dilation and curettage. AFP concentrations in the evacuated products of conception (POC) of the dilation and curettage group, vaginal blood AFP concentrations of the other three groups, and maternal serum AFP (MS-AFP) concentrations of the four groups were evaluated. The median concentration ratio of AFP in vaginal blood to MS-AFP was 24.5 (5.1–8,620) for the missed/incomplete miscarriage group, whereas the ratio of AFP in POC to MS-AFP was 957 (4.6–24,216) for the dilation and curettage group. The AUC was 0.96 at a cutoff ratio of 4.3 with 100% sensitivity and approximately 87% specificity for distinguishing IUP failure from threatened miscarriage. A 73.3% sensitivity and 100% specificity were acquired if the cutoff ratio was 13.4. A [AFP]_vaginal blood_/[AFP]_maternal serum_ ratio ≤ 4.3 indicated a threatened miscarriage. When the ratio was > 13.4, IUP failure occurred. The ratios for the threatened miscarriage group and the cerclage group were 1.2 (0.4–13.4) and 1.01 (0.7–1.5), respectively. However, in cases of severe maternal hemorrhage, AFP from fetal tissue may be greatly diluted, which can lead to false-negative results. The author suggested that the [AFP]_vaginal blood_/[AFP]_maternal serum_ ratio is a novel and time-saving indicator for the diagnosis of a failed IUP.

### Adverse pregnancy outcomes (APOs)

APOs are the major causes of fetal, neonatal, and maternal death and complications, which include preterm birth, preeclampsia, stillbirth, and small for gestational age (SGA) (56). Yefet et al. elaborated the predictive efficiencies of second-trimester MS-AFP and β-hCG for APOs. A total of 14,949 singleton deliveries from 2005 to 2012 were divided into three groups according to MoM ≥ 2: 0 elevated markers group, 1 elevated marker group, and 2 elevated markers group (57). The rate of APOs was 13% in women with elevated MS-AFP (OR: 1.6, 95% CI: 1.2-2.1) or β-hCG (OR: 1.2, 95% CI: 1.03-1.4). When both markers were elevated, the rate was significantly increased to 31% (compared to the “0 elevated markers” group, OR: 2.9, 95% CI: 2.0-4.3; compared to the “1 elevated marker” group, OR: 2.1, 95% CI: 1.4-3.1). The author further showed that MS-AFP ≥ 1.2 MoM or β-hCG ≥ 1.2 MoM could independently predict APOs via an established prediction model integrated with other maternal parameters. In another study conducted by Hu et al., pregnancy outcomes and MS-AFP levels in the first trimester were retrospectively reviewed (58). Among 3,325 singleton pregnant women, a total of 594 pregnancies experienced APOs, including 362 with SGA infants, 181 with preterm birth, 81 with preeclampsia, and 32 with stillbirth. When the cutoff value of MS-AFP 2.5 MoM was used, higher risks of SGA (OR: 1.90, 95% CI: 1.34-2.69), preterm birth (OR: 2.53, 95% CI: 1.65-3.88), and preeclampsia (OR: 3.05, 95% CI: 1.71-5.43) were observed in women with MS-AFP ≥ 2.5 MoM. Lower neonatal birth weights (p = 0.000) and earlier gestational weeks at delivery (p = 0.004) were highlighted in these women. However, the author found that first-trimester MS-AFP was not a candidate predictor for APOs indicated in this study due to low AUC performance.

Goto et al. performed a meta-analysis (39 cohort studies, 93,968 women and their neonates or fetuses) to investigate the relationship between higher MS-AFP levels and SGA risk (59). The relative risk (RR) of SGA was 2.021 (95% CI: 1.751–2.334) when comparing higher to lower MS-AFP levels. MS-AFP levels contributed to SGA risk in a dose-dependent manner. The RR was 2.962 (95% CI: 2.029–4.324) if an MS-AFP cutoff point ≥ 3 MoM was used, whereas the RR was 1.575 (95% CI: 1.245–1.993) if an MS-AFP cutoff point < 1 MoM was used. The author strongly recommended monitoring MS-AFP to identify women at high risk of SGA and to reduce mortality and morbidity.

## Fetal diseases

### Hypospadias

Fetal hypospadias is a common congenital malformation of the male external genitalia that affects 0.4–8.2 of 1000 live male babies (60). In a pioneering multicenter case-control study, Chen et al. divided pregnant women with singleton gestations into two groups: 62 cases without fetal hypospadias and 69 cases with fetal hypospadias at gestational age between 15 and 20 + ^6^ weeks (61). The author found that the MS-AFP MoM was higher [1.14 (0.65-3.40)] in the study group than [0.96 (0.55-1.94)] in the control group (p = 0.005). The AUC of fetal hypospadias predicted by MS-AFP was 0.644 (95% CI: 0.550–0.737, p = 0.005), and the best cutoff value of MS-AFP for fetal hypospadias screening was 0.945 MoM, with a sensitivity of 73.9%, a specificity of 48.4%, and a Youden index of 0.223. The AUC of fetal hypospadias predicted by MS-AFP and β-hCG was 0.700 (95% CI: 0.610–0.789, p < 0.001), with a sensitivity of 55.1% and a specificity of 85.5%. The author suggested the optimal combination of MS-AFP and β-hCG, rather than each marker alone, for fetal hypospadias prediction during the mid-trimester.

### Neural tube defects (NTDs)

NTDs are serious congenital malformations (62). The neuroepithelium is exposed to amniotic fluid if neural tube closure is incomplete, leading to neuron deficiency and degeneration (63). At present, at least two cost-effective parameters, AFP (MS-AFP and amniotic fluid AFP) and maternal age, are used in combination for risk estimates of NTDs (64, 65). However, detecting AFP in amniotic fluid requires invasive amniocentesis, which may cause intrauterine infection or spontaneous abortion (66). MS-AFP is relatively feasible for prenatal screening. Dong et al. investigated more optimal maternal serum biomarkers for noninvasive NTDs screening (67). Serum specimens from 38 pregnant women carrying healthy fetuses and 37 pregnant women carrying NTDs fetuses were collected. A support vector machine (SVM) classifier was used to analyze small samples to establish disease prediction models (68). The SVM model of the complement factors (identified by differential proteomic analysis) had an accuracy rate of 62.5%, a specificity of 67%, and a sensitivity of 60%. In this study, MS-AFP was approximately 400 ng/mL in the spina bifida aperta group. The accuracy rate, specificity, and sensitivity of the SVM model of MS-AFP were 62.5%, 50%, and 75%, respectively. The accuracy of the SVM model could be further increased to 75% by the combination of MS-AFP and the complement factors, and the specificity and sensitivity were also increased to 75%. Notably, MS-AFP is elevated during early embryonic development, and the process of closed NTDs does not release additional AFP. This may cause a missed diagnosis (69).

## Discussion

**Figure 1:**
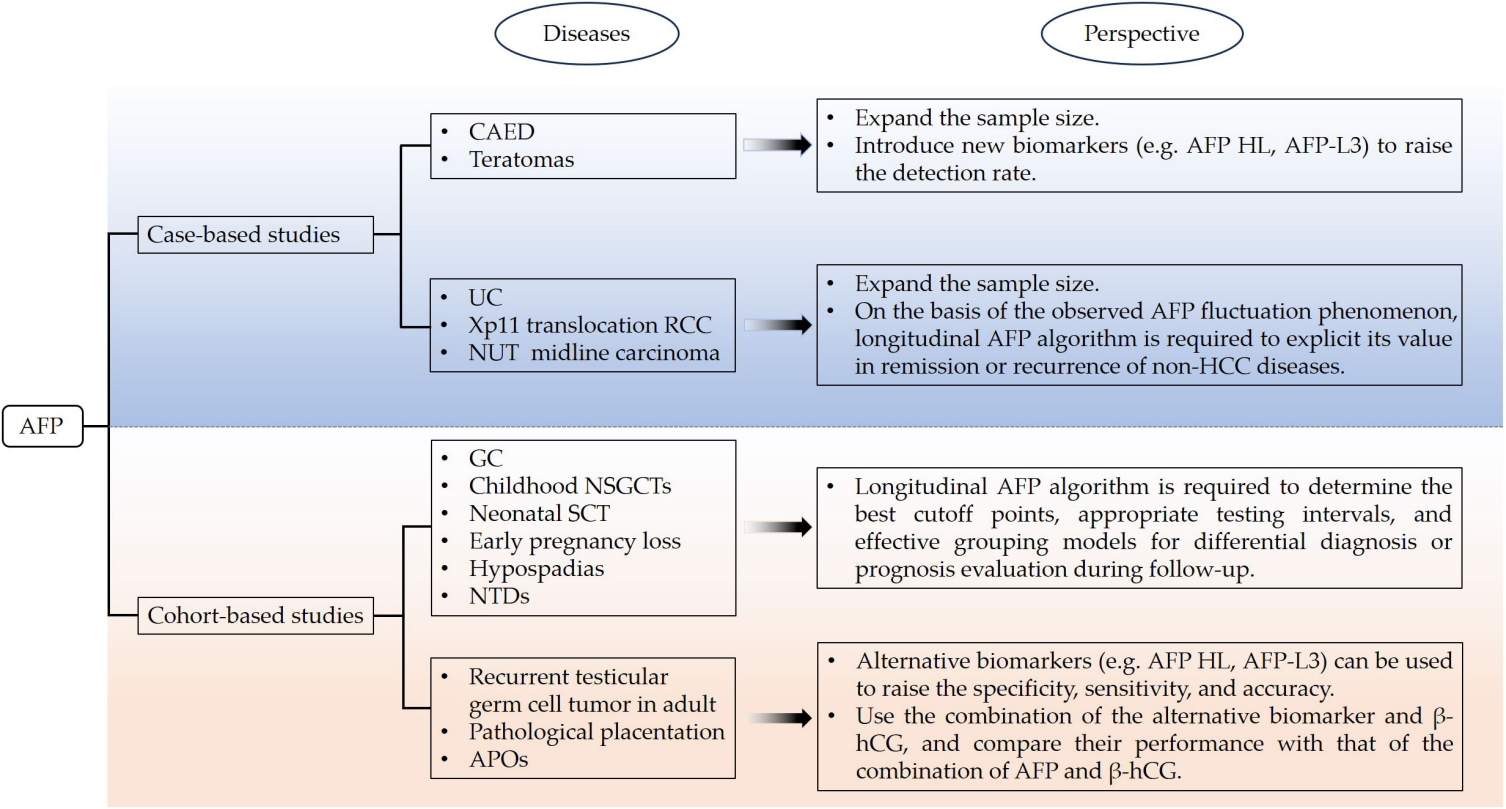
Future research theme for the clinical applications of AFP in non-HCC diseases.

As mentioned above, AFP has alternative clinical applications that are not limited to HCC diagnosis (**Table 1**). With the deepening of research, the accuracy, sensitivity, and specificity of AFP as a guideline-recommended biomarker for HCC surveillance are being challenged (70). Likewise, diagnostic conclusions of non-HCC diseases might be drawn quite differently according to variables such as the usage of serum AFP cutoff levels, degree of tissue dedifferentiation, and capability of retrodifferentiation. Therefore, research should be continuously promoted according to different study categories as well as non-HCC diseases (**Figure 1**). It has been reported that malignant hepatocytes produce a subfraction of AFP, AFP-L3 (71). The ratio of AFP-L3 to total AFP was more sensitive for small HCC lesions, which could be overwhelmed by serum AFP detection alone or ultrasound imaging (72). It is worthwhile to investigate whether AFP-L3, if any, plays a role in CAED, teratomas, recurrent testicular germ cell tumor in adult, pathological placentation, or APOs to improve detection efficiency alone or in combination with established biomarkers (e.g., β-hCG) (**Figure 1**). In a follow-up study conducted by Choi et al., they found that the AUC of the serial AFP level-based algorithm for HCC diagnosis was higher than that of AFP alone at a fixed threshold (0.94 vs. 0.86, p <0.0005) (73). The performance and effectiveness of the longitudinal AFP algorithm in non-HCC diseases needs to undergo intensive validation in retrospective and prospective studies. For the observed AFP fluctuation in several case reports, the longitudinal AFP algorithm can be used to monitor disease progression in cohort studies of UC, Xp11 translocation RCC, and NUT midline carcinoma in the future. The longitudinal AFP algorithm will also favor differential diagnosis or prognosis evaluation in GC, childhood NSGCTs, neonatal SCT, early pregnancy loss, hypospadias, and NTDs in which serum AFP levels are only detected on admission or before treatment (**Figure 1**).

**Table 1:** Overview of diverse applications of AFP.

Conditions	Number of patients	Description of the clinical features	Clinical relevance	References
Tumors				
GC	97 AFP-positive cases in a total of 3,034 GC patients	1. AFP, age, and pathologic tumor stage were predictive for OS (p < 0.0001). 2. RFS was worse in AFPP-GC patients (p = 0.002). 3. Recurrence was more frequent in AFPP-GC patients (34.5%) than in AFPN-GC patients (16.9%). 4. Five-year survival rate was lower in AFPP-GC patients (57.9%) than in AFPN-GC patients (76.1%). 5. Five-year RFS rate was lower in AFPP-GC patients (53%) than in AFPN-GC patients (76%).	A preoperative elevation of AFP (> 10 µg/L) is an independent poor prognostic predictor of OS and RFS.	Reim et al. (11)
CAED	Five CAED cases from 1,666 patients with colorectal carcinoma and one AFP-positive CAED case	IHC staining showed one CAED case was positive for AFP.	A characteristic marker for hepatoid components	Murakami et al. (14)
	One case	IHC staining revealed the tumor cells from the liver metastases were immunoreactive for markers including AFP, GPC-3, and SALL4.		Abada et al. (15)
Xp11 translocation RCC	Two cases	1. Serum AFP levels significantly dropped after nephrectomy. 2. AFP was diffusely positive in tumor cells.	When serum AFP is elevated and a patient does not have explainable liver or germ cell tumors, a search for RCC is recommended.	Zhang and Wang (17) Shim et al. (18)
UC	One case	1. Serum AFP levels were closely related to tumor burden. 2. IHC examination of the primary lesion showed strong staining for AFP.	AFP is a tumor marker for anti-PD-1 therapy response.	Melms et al. (24)
Recurrent testicular germ cell tumor in adult	Systematic review including nine studies	1. The specificities of AFP was high (95-100%). 2. The sensitivities of AFP ranged from 36% to 100% when used alone or in combination with β-hCG.	More evidence is required to guide testing with biomarkers for recurrence surveillance.	Nicholson et al. (25)
Childhood NSGCTs	239 NSGCTs patients including 92 boys and 147 girls (median age: 3 years, range = 0.1-18 years)	1. A higher risk of progression/recurrence was observed in patients who had slower serum AFP decline. 2. PFS was significantly poorer when the difference between observed and expected AFP decline AUC increased.	The difference between O-E AUCs is of prognostic value.	Fresneau et al. (28)
Neonatal SCT	55 neonates with nonrecurrent SCT and 10 neonates with recurrent SCT	1. Serum AFP HL was correlated with age using the formula T_HL_= 0.0597 × days + 6.1643 in nonrecurrent SCT patients. 2. Serum AFP HL was correlated with age using the formula T_HL_= 0.1196 × days −0.0633 in recurrent SCT patients. 3. Serum AFP HLs was calculated using the formula T_HL_= 0.0433 × days +8.9339 in immature SCT. 4. Serum AFP HLs was calculated using the formula T_HL_= 0.0671 × days + 4.3912 in mature SCT.	When being in proportion to the age, AFP HL is longer in recurrent SCT as well as in immature SCT.	Nam et al. (35)
NUT midline carcinoma	One case	1. Serum AFP level declined after two chemotherapy cycles along with attenuation of the superior vena cava syndrome. 2. Serum AFP level increased when disease progressed.	Measurement of serum AFP during treatment to monitor disease course is suggested.	D’Ambrosio et al. (41)
Gynecological and Obstetric diseases				
Teratomas	One case	Serum AFP levels were persistently high in a patient with recurrence of benign teratomas in multiple locations.	–	Caposole et al. (44)
Pathological placentation	307 deliveries including 64 cases with placenta accreta, 66 cases with placenta previa, 17 cases with both, and 153 cases with normal placentation	1. Patients with pathological placentation had a higher median AFP and β-hCG MoM. 2. For pathological placentation prediction, the AUC of AFP was 0.573 (95% CI: 0.515-0.630, p < 0.0274) with 46% specificity and 71% sensitivity at a cutoff of 0.99 MoM. 3. When combined with β-hCG, the AUC was 0.668 (95% CI: 0.611-0.721, p < 0.0001) with 64% specificity and 63% sensitivity for pathological placentation prediction. 4. The 75^th^ percentile MoM cutoff of AFP or β-hCG or both led to a 2.27 OR (95% CI: 1.42-3.63) for pathological placentation.	A diagnosis marker for pathological placentation during second trimester	Berezowsky et al. (49)
	One case	High serum AFP level was seen in central placenta previa accompanied by intermittent bleeding and placental necrosis.	The combination of AFP and ultrasound imaging may be used to assess the status of placenta previa.	Yu et al. (51)
Early pregnancy loss	78 patients including 31 cases with missed/incomplete miscarriage, 15 cases with threatened miscarriage, 9 cases with cerclage, and 23 cases with dilation and curettage	1. The median concentration ratio of AFP in vaginal blood to MS-AFP was 24.5 (5.1–8,620) for the missed/incomplete miscarriage group. 2. The median concentration ratio of AFP in POC to MS-AFP was 957 (4.6–24,216) for the dilation and curettage group. 3. The AUC was 0.96 with 100% sensitivity and 86.7% specificity at a cutoff ratio 4.3 for distinguishing IUP failure from a threatened miscarriage. 4. If cutoff ratio was 13.4, the sensitivity and specificity were 73.3% and 100%, respectively. 5. [AFP]_vaginal blood_/[AFP]_maternal serum_ ratio ≤ 4.3, a threatened miscarriage occurred. [AFP]_vaginal blood_/[AFP]_maternal serum_ ratio > 13.4, IUP failure occurred.	[AFP]_vaginal blood_/[AFP]_maternal serum_ ratio is a novel and time-saving indicator for IUP failure diagnosis.	Mor et al. (55)
APOs	14,949 singleton deliveries in second-trimester	1. The rate of APOs was 13% in women with elevated MS-AFP (OR: 1.6, 95% CI: 1.2-2.1) or β-hCG (OR: 1.2, 95% CI: 1.03-1.4). 2. The rate of APOs was 31% in women with both elevated markers. 3. MS-AFP ≥ 1.2 MoM or β-hCG ≥ 1.2 MoM independently predicted APOs with additional maternal characteristics.	1. Elevation of both MS-AFP and β-hCG are markers for APOs. 2. MS-AFP, β-hCG, and additional maternal characteristics can predict high risk of APOs.	Yefet et al. (57)
	594 women experienced APOs in first-trimester including 362 cases with SGA, 181 cases with preterm birth, 81 cases with preeclampsia, and 32 cases with stillbirth	1. In women with MS-AFP ≥ 2.5 MoM, higher risks of SGA (OR: 1.90, 95% CI: 1.34-2.69), preterm birth (OR: 2.53, 95% CI: 1.65-3.88), and preeclampsia (OR: 3.05, 95% CI: 1.71-5.43). 2. In women with MS-AFP ≥ 2.5 MoM, lower neonatal birth weights (p = 0.000) and earlier gestational weeks at delivery (p = 0.004) were indicated.	First-trimester MS-AFP cannot predict APOs.	Hu et al. (58)
	A meta-analysis containing 39 cohort studies and 93,968 women and their neonates or fetuses	1. When compared higher to lower MS-AFP levels, the RR of SGA was 2.021 (95% CI: 1.751–2.334). 2. RR was 2.962 (95% CI: 2.029–4.324) when MS-AFP cutoff point ≥ 3 MoM. 3. RR was 1.575 (95% CI: 1.245–1.993) when MS-AFP cutoff point < 1 MoM.	MS-AFP level contributes SGA risk in a dose-dependent manner.	Goto et al. (59)
Fetal diseases				
Hypospadias	A multicenter case-control study including 69 pregnant women with fetal hypospadias and 62 pregnant women without fetal hypospadias in mid-trimester.	1. MS-AFP MoM was higher in the fetal hypospadias group compared to that in the control group (1.14 vs. 0.96, p = 0.005). 2. The AUC of fetal hypospadias predicted by MS-AFP was 0.644 (95% CI: 0.550–0.737, p = 0.005). 3. When cutoff value 0.945 MoM was used for fetal hypospadias screening by MS-AFP, the sensitivity, specificity, and Youden index were 73.9%, 48.4%, and 0.223, respectively. 4. The AUC of fetal hypospadias predicted by MS-AFP and β-hCG was 0.700 (95% CI: 0.610–0.789, p < 0.001) with a sensitivity of 55.1% and a specificity of 85.5%.	Combination of mid-trimester MS-AFP and β-hCG levels can be used as a marker for fetal hypospadias screening.	Chen et al. (61)
NTDs	38 pregnant women carrying healthy fetuses and 37 pregnant women carrying NTDs fetuses.	1. The SVM model of MS-AFP had an accuracy rate of 62.5%, a specificity of 50%, and a sensitivity of 75%. 2. The SVM model of MS-AFP and the complement factors showed 75% accuracy rate, 75% specificity, and 75% sensitivity.	A SVM model based on MS-AFP and complement factors is a promising noninvasive method for accurate diagnosis of prenatal NTDs.	Dong et al. (67)

Notably, the reference interval (RI) of AFP needs to be determined in the pediatric population with a focus on data sources, child age, and the effects of gestational age, sex, and birth weight (74–76). The high variability of the currently available AFP RIs is a major limitation, particularly in infants under 6 months of age. New research with defined infant serum AFP RIs may improve the interpretation of laboratory results. Clinical models and algorithms based on absolute serum AFP HLs and concentrations will benefit pediatric diagnosis.

## Conclusion

AFP is a characteristic biomarker of CAED, RCC, pathological placentation, early pregnancy loss, and NTDs. Serum AFP fluctuation can be used for monitoring the disease course, risk evaluation, and treatment response and can determine clinical outcomes in patients with GC, urothelial carcinoma, childhood NSGCTs, neonatal SCT, NUT midline carcinoma, and APOs. When combined with β-hCG, AFP has greater reference value in the diagnosis and risk prediction of APOs as well as in fetal hypospadias screening. It may underline a future direction to broad AFP utilization in combination with subfractions of AFP, other serum parameters, and imaging examination approaches in non-HCC diseases.

## Author contributions

CM, YJ, and YW designed the mini review, drafted the work, and approved the content. HX and JZ revised it critically for important intellectual content and approved the content. All authors contributed to the article and approved the submitted version.
